# Perceptuomotor compatibility effects in vowels: Beyond phonemic identity

**DOI:** 10.3758/s13414-020-02014-1

**Published:** 2020-03-31

**Authors:** Payam Ghaffarvand Mokari, Adamantios Gafos, Daniel Williams

**Affiliations:** 1 Department of Linguistics, University of Potsdam, Potsdam, Germany; 2 Haskins Laboratories, Yale University, New Haven, CT USA

**Keywords:** speech perception, speech production, psycholinguistics

## Abstract

Perceptuomotor compatibility between phonemically identical spoken and perceived syllables has been found to speed up response times (RTs) in speech production tasks. However, research on compatibility effects between perceived and produced stimuli at the subphonemic level is limited. Using a cue–distractor task, we investigated the effects of phonemic and subphonemic congruency in pairs of vowels. On each trial, a visual cue prompted individuals to produce a response vowel, and after the visual cue appeared a distractor vowel was auditorily presented while speakers were planning to produce the response vowel. The results revealed effects on RTs due to phonemic congruency (same vs. different vowels) between the response and distractor vowels, which resemble effects previously seen for consonants. Beyond phonemic congruency, we assessed how RTs are modulated as a function of the degree of subphonemic similarity between the response and distractor vowels. Higher similarity between the response and distractor in terms of phonological distance—defined by number of mismatching phonological features—resulted in faster RTs. However, the exact patterns of RTs varied across response–distractor vowel pairs. We discuss how different assumptions about phonological feature representations may account for the different patterns observed in RTs across response–distractor pairs. Our findings on the effects of perceived stimuli on produced speech at a more detailed level of representation than phonemic identity necessitate a more direct and specific formulation of the perception–production link. Additionally, these results extend previously reported perceptuomotor interactions mainly involving consonants to vowels.

Previous research has found that the speed of producing a spoken response and in some cases the phonetic parameters of the produced response can be modulated systematically by stimuli perceived while planning that response (Adank, Nuttall, Bekkering, & Maegherman, 2018; Galantucci, Fowler, & Goldstein, 2009; Kerzel & Bekkering, 2000; Roon & Gafos, 2015; Tobin, Hullebus, & Gafos, 2018). These modulations have been referred to as “perceptuomotor effects” because they derive from the influences of perceived stimuli on the production of the intended response. As such, these effects enable elaboration of the perception–production link, the nature of the representations involved in that link, and the likely computational principles that subserve its functioning (Roon & Gafos, 2016).

In exploring how the speech production and perception systems interact during online processing, an experimental paradigm that requires concurrent use of both systems has been particularly revealing. This paradigm originates outside of speech, from studies on manual action addressing the general question of how observing a movement affects executing that movement along with its allied stimulus–response compatibility field (Hommel, Müsseler, Aschersleben, & Prinz, 2001; Kornblum, Hasbroucq, & Osman, 1990) with antecedents in earlier work by Paul Fitts and colleagues (Fitts & Deininger, 1954; Fitts & Seeger, 1953). In the context of speech perception and production, this paradigm is instantiated by the so-called cue–distractor task. Each trial of this task begins by the participant being prompted (e.g., by a visual cue) to produce a spoken response (usually a single syllable) and around the same time, while the participant is planning to produce their spoken response, a distractor stimulus (an audio or video speech recording) is presented. Across trials, the relation between the cued response and the distractor is varied so that the two (mis)match in terms of some set of parameters under the control of the experimenter. The main finding is that the degree of similarity (in terms of this set of parameters) between the perceived stimulus and the cued spoken response modulates the speed with which the response is produced. We illustrate this main finding with some examples.

Kerzel and Bekkering (2000), who were the first to employ the cue–distractor task in speech, found that congruency between a cued response syllable and a silent video of a speaker mouthing another syllable speeds up providing the cued response. In one experiment, participants learned to produce a /ba/ or /da/ syllable when cued with one of two possible symbols (## or &&). At some time before presentation of the symbols, a video of a face producing a /b/-initial or /d/-initial syllable was shown. Reaction times for the required responses were faster if the symbol cued the response consistent with the video. In interpreting this result, Kerzel and Bekkering (2000) proposed that seeing a visual gesture activates the motor codes to produce that gesture. This leads to the interference effects observed in their experiments. That is, observing a movement involving a bilabial closure leads to faster reaction times when the response is /ba/, because the motor codes of the required response are congruent with motor codes activated from the visual stimulus. If, instead, the motor codes activated from the visual stimulus are not congruent with the required response (e.g., seeing a video of a face producing /d/-initial syllable while cued to respond by saying /ba/), the incompatible response is activated, but must be inhibited, leading to slower reaction times.

Galantucci et al. (2009) argued that using auditory instead of visual distractors would provide an even stronger test for the immediacy of the link between speech perception and production, and specifically test for the activation of motor codes from auditory input. This is because motor codes activated from a visual stimulus (due to orofacial information about articulatory action) would not be available in the same way for an auditory stimulus and must somehow be transduced from the distal acoustic signal. Therefore, Galantucci et al. (2009) set out to replicate Kerzel and Bekkering’s (2000) findings by using auditory materials and found that RTs speed up when participants hear the same syllable that they were preparing to say (e.g., /ba/–/ba/) compared with when they hear a syllable beginning with a consonant using a different articulator (e.g., /ba/–/da/).

Whereas the results from Galantucci et al. (2009) and Kerzel and Bekkering (2000) concern perceptuomotor compatibility effects for identical versus different consonants, other studies have shown that such effects can be demonstrated for properties more fine-grained than phonemic identity. Thus, building on Galantucci et al.’s (2009) findings, Roon and Gafos (2015) found that RTs speed up when the initial consonants of the cued response and auditory distractor share the same voicing (e.g., /ɡa/–/ba/) compared with different voicing (e.g., /ka/–/ba/) or share the same articulator (e.g., /pa/–/ba/) compared with different articulators (e.g., /ta/–/ba/). In each case, the cued response and distractor stimulus are always phonemically different. Congruency in terms of the same voicing (but different articulator) or same articulator (but different voicing) resulted in the speeding up of responses compared with incongruency (that is, both voicing and articulator being different). This result highlights that congruency between a response and a distractor is not only a matter of same versus different consonants; crucially, subphonemic properties (below the level of phonemic category and in particular here voicing and articulator, two key parameters of phonological contrast; Chomsky & Halle, 1968) play a role in modulating perceptuomotor effects (for parallel work in the nonspeech domain aiming to identify parameters over which congruency is expressed, see Brass, Bekkering, and Prinz, 2001; Stürmer, Aschersleben, & Prinz, 2000).

Notably, the studies examining perceptuomotor effects we have examined above have focused on consonants (Galantucci et al., 2009; Kerzel & Bekkering, 2000; Klein, Roon, & Gafos, 2015; Roon & Gafos, 2015; Tobin et al.*,*^2018^). However, one recent study indicates that perceptuomotor effects do occur with vowels at the phonemic level. Adank et al. (2018) examined perceptuomotor effects with the syllables *heed* and *hood*, which exhibit a phonemic difference only in their vowels (/i/ and /ʊ/), using the cue–distractor task. The task involved participants producing spoken responses to a written *heed* or *hood* prompt in the presence of a background distractor (heed or hood) presented in video, audio, or audio-visual modalities. Like for consonants, Adank et al. (2018) observed faster RTs on congruent trials in all modalities (e.g., when the response and distractor were both *heed* or both *hood*) compared with incongruent trials (e.g., when the response was *hood* and the distractor was *heed* or vice versa). Critically, as the study used a single pair of vowels, it was not possible to determine whether congruency between a response and distractor comprises subphonemic properties—that is, if and how properties of vowels beyond same versus different phonemes may also modulate RTs. This is the issue we address in our study.

Our first hypothesis is that phonemically identical response–distractor pairs of vowels (e.g., /e/–/e/ or /u/–/u/) will result in faster RTs than phonemically different pairs (e.g., /e/–/u/ or /u/–/i/) in the cue–distractor task. Our second hypothesis considers congruency beyond phonemic (non-)identity. Specifically, extrapolating from perceptuomotor effects at a subphonemic level for consonants (Roon & Gafos, 2015), our second hypothesis is that distractor vowels which are more like the response vowel will speed up RTs compared with distractor vowels, which are more different. One of the similarity measures we will employ to express the notion of “the distractor vowel being more or less like the response vowel” is defined by the number of phonological features that a pair of vowels differ on using standard featural presentations (Chomsky & Halle, 1968). Introducing this measure allows us to also highlight one of the key motivations for the present study.

It is not at present clear whether previously found perceptuomotor compatibility effects at subphonemic levels for consonants (Roon & Gafos, 2015) should extend to vowels. The two parameters of consonant classification for which perceptuomotor effects have been investigated are the main articulator used to produce the consonant and its voicing. Let us illustrate with an example. In Roon and Gafos’s (2015) experiment on consonant articulator, distractors never matched responses in voicing, but had an articulator that was either congruent or incongruent with the response. For instance, when the response is /pa/ and the distractor is /ba/, both response and distractor begin with a closing action of the lips and are therefore considered congruent. On the other hand, the response–distractor pair /pa/–/da/ is considered incongruent because the response involves the lips closing while the distractor involves a different organ—the tongue—making a closure at the alveolar ridge. The perceptuomotor effect here consists in the speeding up of the response when the perceived distractor implicates the same organ as the response compared with when it does not. Asking whether a parallel effect can be demonstrated for vowels turns out not to be a straightforward question. Consonants and vowels are conventionally characterized in different ways (Chomsky & Halle, 1968). Whereas primary among the parameters that characterize consonants are the main articulator or organ used to effect the constriction required for the consonant (lips for /p/ vs. tongue body for /ɡ/) and aspects of the coordination of that constriction with laryngeal action (that is, voicing for languages like English and German with voiceless /p/ and voiced /b/), vowels are not so described. At least in the languages that have provided the main evidence for perceptuomotor effects thus far, all vowels are voiced and all are produced with different characteristic postures of the same set of organs—namely, the tongue body and the lips. That is, unlike for the consonants /b/ and /ɡ/, where /b/ does not implicate the tongue body and /ɡ/ does not implicate the lips, any two vowels implicate the same organs (and are produced with the vocal folds in their modal voicing state).

A standard way to describe linguistically relevant differences among vowels (Chomsky & Halle, 1968) is via a set of binary features such as [±high], [±low], [±back], [±round], [±tense] with each feature specifying a value for a vowel along the abstract dimensions of height, backness, roundness, and tenseness. The relevant dimensions are abstract because the relation between a feature and its physiological correlates in terms of vocal tract shape and corresponding acoustics is complex (Ladefoged, 1980). What is relevant for our purposes is that any such set of features naturally gives rise to a similarity metric among vowels based on these features. For example, the phonemes /o/ and /u/ are identical except for the values of just one feature (/o/ is [−high] and /u/ is [+high]), while the phonemes /i/ and /u/ are identical except for the values of two features (/i/ is [−round] and [−back] and /u/ is [+round] and [+back]) and so on. In this way, /i/ and /u/ may be described as more different from one another than /o/ and /u/ because the former pair displays a greater number of featural differences than the latter pair. Quantifying featural differences among pairs of phonemes in such a manner has a long tradition of use in experimental linguistics and psycholinguistics to index subphonemic similarity (Bailey & Hahn, 2005; Frisch, Pierrehumbert, & Broe, 2004; Monaghan, Christiansen, Farmer, & Fitneva, 2010; Scharinger, Domahs, Klein, & Domahs, 2016; Schepens, van Hout, & Jaeger, 2020; Wilson & Obdeyn, 2009).

Overall, uncovering the dimensions of representations over which perceptuomotor effects occur (between a perceived distractor and a produced response) would help in outlining the minimal levels of information involved in the link between speech perception and production. This informs theories of speech perception, theories of speech production, and, if perceptuomotor effects can be demonstrated, theories of how properties of a perceived stimulus affect production of speech. It remains unclear, for example, if and how differences in the nature of auditory and phonetic memory codes activated in perceiving different classes of sounds (e.g., consonants versus vowels; Grabski et al., 2013; Obleser, Leaver, VanMeter, & Rauschecker, 2010; Pisoni, 1973), influence concurrent speech production. Some speech production models assign roles for subphonemic parameters in the process of speech production (e.g., Dell, Juliano, & Govindjee, 1993), while other theories do not assign important roles for subphonemic features in the process of planning articulation (e.g., Roelofs, 1997). Given the differences on specificity of the representations in different speech production models, the findings of this study would have implications for these models (e.g., Bohland, Bullock, & Guenther, 2009; Dell et al., 1993; Roelofs, 1997). More generally, the findings of this study would shed light on how perception and production are related as well as the kinds of representations that may be involved in the link between these two domains.

## Method

### Participants

Participants were 38 native speakers of Standard German with a mean age of 26 years (*SD* = 5.5). All participants were students in the University of Potsdam, and all reported normal hearing and no history of speech problems. Participants gave informed consent and received course credit or payment for their participation.

### Stimuli

The two response vowels were /e/ and /u/, and the four distractor vowels were /e/, /i/, /o/, /u/. This choice of responses and distractors allows us to generate response–distractor pairs with the featural distances of 0, 1, 2 and 3 (as will be made explicit in the forthcoming Table 2). For both responses /e/ and /u/, featural distances to their paired distractors vary in the same range, from a minimal distance of 0 (for response /e/– distractor /e/ and response /u/– distractor /u/) up to a maximal distance of 3 (for response /e/– distractor /u/ and response /u/– distractor /e/). In this first systematic assessment of perceptuomotor compatibility effects at a subphonemic level for vowels, these two response vowels offer the minimal set of vowels for which the featural distance can be varied to the same extent, thus rendering our experimental session feasible with respect to the time required to complete a session. Including any other response vowel would not increase resolution of the featural distance metric from that obtained by using /e/ and /u/.

Instances of the four distractor vowels in consonant–vowel–consonant syllables (/d–vowel–k/) were recorded by a 36-year-old female native speaker of Standard German in an anechoic chamber using a unidirectional microphone (Audiotechnica 4028a) connected to an M-Audio Delta Audiophile sound card via a Phonic MM 1705A mixer. The recordings were saved in a single channel sampled at 44.1 kHz with 16 bits in wave-file format. From each of these four syllables, vowel-only stimuli were created by extracting the stable vowel portion (free of noticeable formant/pitch deviations) from each syllable. All four so-extracted vowels tokens were then edited to be 150 ms and normalized for peak intensity. A fade in/out was applied to all tokens in order to prevent click-like sounds caused by high amplitudes at the beginnings and ends of the signals. The resulting sound files served as the four distractor vowels in the cue–distractor task. In addition, a 150 ms periodic tone (440 Hz) was synthesized to act as an additional nonspeech distractor.

### Procedure and reaction-time measurements

Participants were seated in front of a monitor in a sound-attenuated booth and wore Sony MDR-AS210 earbuds connected to the laptop running the experiment. The audio output from the laptop (distractors and tone markers) and the participants’ responses were recorded in two separate channels in Audacity (R) recording software (Audacity Team, 2019). The audio output of the laptop was connected both to the earbuds and to the recording PC via a Behringer HA400 preamplifier, a SPL controller (Model 2381), and an AMD high-definition sound card. The participants’ responses were recorded using a YOGA EM-9600 microphone connected to the recording PC via a Schoeps VSR 5 preamplifier connected to the sound card. The recordings were saved in wave format sampled at 44.1 kHz with 16 bits resolution.

Participants were instructed to produce the target vowel as quickly as possible when prompted to do so by the visual cue (say ‘ee’ when they see ** and say ‘uu’ when they see ##) and ignore what they heard from the earbuds. Before the beginning of the experiment, the experimenter provided participants with example pronunciations to demonstrate the intended German target vowels. Each trial started with a fixation point (+) that appeared in middle of a black screen for 500 ms, after which the cue symbol (** or ##) appeared in the middle of the screen. The cue stayed on-screen until a response was detected. Participants were given a short practice block (20 trials) before beginning the experiment. The first 10 practice trials were without a distractor, and the next 10 included both distractor and no-distractor trials. Experimental trials were divided into three blocks so that participants could have a break between blocks. Each block started with an instruction screen providing the symbol–vowel pairs in a light grey Ubuntu font with 78-point font size on a black background. We used the orthographic representations ‘ee’ and ‘uu’ (with double letters) to make sure that participants did not confuse the intended target vowels with the German short counterparts (/ɛ/ and /ʊ/).

The distractors were the /e/, /u/, /i/, and /o/ vowels and the tone distractor described above and stimulus presentation was handled by Psychtoolbox 3.0 (Kleiner et al., 2007) implemented in MATLAB (The MathWorks, Natick, MA). The tone distractor corresponded to the nonspeech distractor condition of previous cue–distractor studies (e.g., Galantucci et al., 2009; Roon & Gafos, 2015) to index the general effect of an auditory distractor. All distractors were presented with a stimulus onset asynchrony (SOA) of 150 or 200 ms after the visual cue. Each response–distractor combination was presented 30 times at each of the two SOAs, yielding 600 trials (5 distractors × 2 SOAs × 2 responses × 30 repetitions). For RT experiments, Brysbaert and Stevens (2018) recommend a minimum of 1,600 observations per condition. As each response–distractor pair was presented a total of 60 times (2 SOAs × 30 repetitions) per participant, at least 27 participants were needed to reach this minimum number of observations for each response–distractor pair. Additionally, 120 no-distractor trials were included to index the general effect of distractors compared with trials without any distractor. These trials were the same as the distractor trials except that no distractor was presented after the appearance of the visual cue. The total number of trials per participant was thus 720 trials and the order of trials was pseudorandomized across participants. The experiment session lasted about 35 minutes for each participant.

**Fig. 1:**
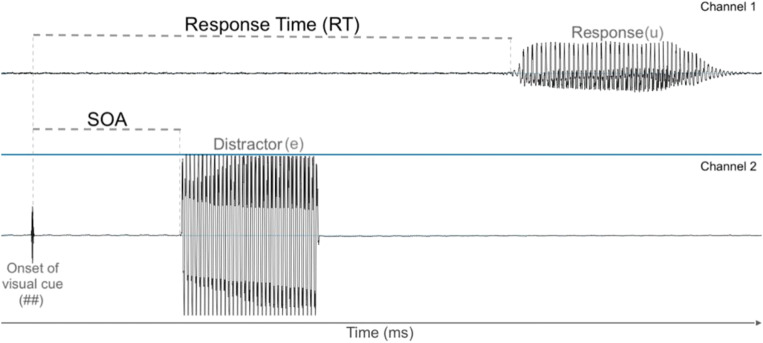
An example of an RT measurement for a trial where the response is the vowel /u/ and the distractor is vowel /e/. The top audio channel shows the participant’s spoken response. The bottom audio channel shows the time line for the presentation of visual cue and the auditory distractor. The short vertical marker in the second channel is synchronous with the onset of the presentation of the visual cue. Stimulus onset asynchrony (SOA) is the latency between that onset and the onset of the auditory distractor (here, 150 ms). RT was calculated as the latency between (the time stamp of) the onset of the spoken response minus (the time stamp of) the onset of the visual cue

Spoken responses were recorded in one audio channel while a separate audio channel recorded distractors and 2.5-ms tone markers indicating the time at which the visual cues appeared. RTs were manually measured as the latency between the center of the 2.5-ms tone in the second channel (which was synchronous with the presentation of the onset of the visual cue ## or **) and the start of waveform fluctuations in the first channel corresponding to participants’ spoken response (see Fig. 1). Labeling of the audio files was conducted in the computer program Praat (Boersma & Weenink, 2019). For every trial, boundaries were placed on the tone marker and at the beginning of the spoken response vowel using a wide time window of around 1 second. Subsequently, these boundaries were moved to precise locations using a 20 ms window. The research assistants who were responsible for manually labeling the audio recordings and obtaining RT measurements were not aware of the experimental conditions.

### Statistical analyses

A set of linear mixed-effect models were fitted for the analysis. In each model, the dependent variable was log-transformed RTs (log RTs). “Participant” and “trial” were included as random effects, and “distractor condition” was included as fixed factor. Following Roon and Gafos (2015), other fixed factors were included to control for nonlinguistic effects that are not of experimental interest and are expected to influence RTs in the cue–distractor task. In other words, these ‘nonlinguistic factors’ are modelled as control variables in our statistical assessment based on their effects on the dependent variable reported in prior cue–distractor studies (Galantucci et al., 2009; Kerzel & Bekkering, 2000; Roon & Gafos, 2015). The five nonlinguistic factors along with explanations of their inclusion were (1) Log RT of the preceding trial—expected to correlate with the current trial’s log RT; (2) Whether the response on the preceding trial is correct or incorrect—expected to increase the RT of the current trial if incorrect; (3) Whether the cued response is the same as the preceding trial (two treatment levels: same cue or different cue)—expected to decrease the current trial’s RT if the same; (4) SOA (two treatment levels: 150 ms or 200 ms)—longer SOAs are expected to result in longer RTs (Galantucci et al., 2009; Kerzel & Bekkering, 2000; Roon & Gafos, 2015); (5) Trial number—to account for fatigue or habituation over the course of the entire experiment.

## Results

The complete data set comprised 27,360 trials (38 participants × 2 responses × 2 SOAs × 6 distractor conditions × 30 repetitions). A total of 486 trials were excluded due to incorrect responses (e.g., producing an /u/ response when the cue prompted an /e/ response; 1.78% of trials). Also excluded were 1,785 trials (6.52%) on which participants’ responses started less than 100 ms after the onset of the distractor; this is because a distractor cannot evoke perceptuomotor effects if the participant starts producing the response before having had time to perceive most of the 150 ms distractor (Roon & Gafos, 2015). Finally, responses given 750 ms after the start of distractor (314 trials, 1.15%) were excluded based on the assumption that the participants were inattentive on those trials.

**Fig. 2:**
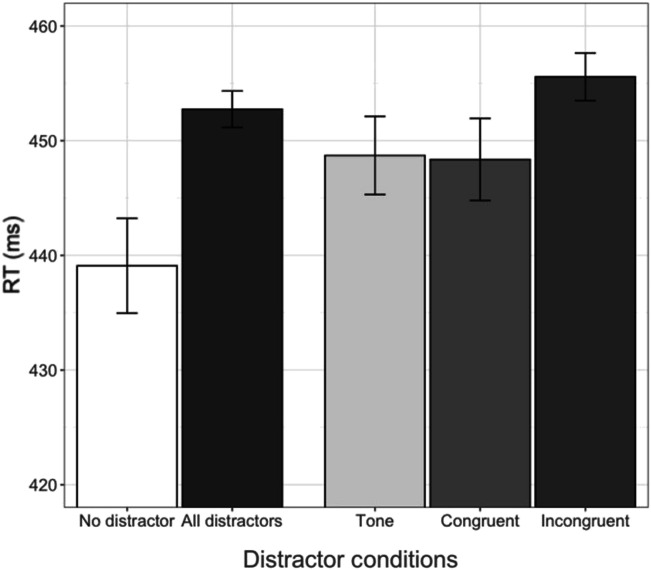
Mean RTs (ms) showing phonemic congruency effects by distractor condition. The “All distractors” bar shows the mean RTs for all trials from every distractor condition (tone, congruent, and incongruent) pooled together. Error bars show 95% confidence intervals. Results are collapsed across SOA (the interactions of SOA and distractor conditions were not significant)

Figure 2 shows the mean RTs (in ms) by distractor condition across both responses. As can be seen, RTs were faster in the no-distractor condition compared with the distractor conditions. This replicates the entirely expected general effect of distractors on providing spoken responses as reported in previous studies (Galantucci et al., 2009; Kerzel & Bekkering, 2000)—namely, the presence of a distractor slows down giving the response. Of course, at issue in our study is how exactly different distractors modulate RTs for the responses.

### Phonemic congruency

The first hypothesis we aim to assess is that when the response and the distractor are phonemically the same (congruent), RTs should be faster than when they are phonemically different (incongruent). Phonemic (non-)identity is the extreme case of (in-)congruency. In the model fitted to test this hypothesis, the fixed effect of experimental interest was distractor condition, which had three treatment levels: tone, congruent, and incongruent. Here congruent means that the response and distractor were phonemically identical (e.g., response = /u/ and distractor = /u/) and incongruent means that they were phonemically different (response–distractors: /u/–/e/, /u/–/i/, /u/–/o/, /e/–/u/, /e/–/o/, and /e/–/i/). Response and the interaction between the response and distractor conditions were also included to examine the generalizability of distractor congruency effect across responses. The interaction between SOA and distractor condition was also included to examine the sensitivity of any distractor congruency effects across SOAs. The random effects structure included by-participant slopes for effects and interaction involving Distractor Condition × SOA × Responses, as these were repeated across participants (Barr, Levy, Scheepers, & Tily, 2013). All nontreatment fixed effects were centered to reduce collinearity (Gelman & Hill, 2007). In total, 20,548 trials were submitted to this analysis.^1^

Table 1 shows the results of the first model. The “ms” column shows log RT values in the “Estimate” column converted into milliseconds, with negative values indicating faster RTs and positive values indicating slower RTs relative to the intercept. An absolute *t* value greater than 2 was considered as significant (Gelman & Hill, 2007). Congruent distractor was the baseline condition, meaning that the coefficients for the tone and incongruent distractors are relative to congruent distractors. RTs were significantly longer when there was an incongruent distractor compared with when there was a congruent distractor (|*t*| = 3.46). The tone distractor did not yield significantly different RTs from congruent distractors. All nonlinguistic effects were significant. The effect of SOA with congruent distractors was significant, indicating that the 200 ms SOA resulted in longer RTs than the 150 ms SOA, and the interactions between SOA and distractor condition were not significant (all |*t*| < 0.23), indicating similar SOA effects for the tone and incongruent distractors. The effect of Response (two levels: /e/ and /u/) was not significant, nor was the interaction between response and distractor.

**Table 1:**
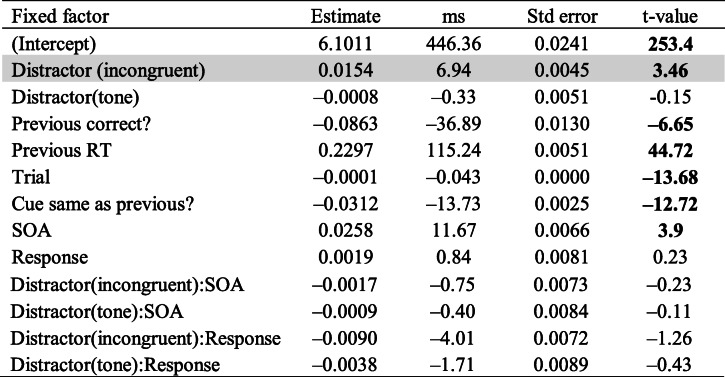
Results of a linear mixed-effect model of log RTs (phonemic congruency effect)

*Note*. The effect of incongruent condition compared with congruent condition is shaded. Boldface *t* values indicate significant predictors in the model (|*t*| > 2)

### Subphonemic congruency

Our second hypothesis concerns the effect of different distractors on RTs. Recall that, on each trial, the two responses /e/ and /u/ were paired with one of the four distractors /i/, /e/, /o/, and /u/. This allows us to address the role of subphonemic features in modulating RTs. Table 2 shows the phonological feature assignments for each response–distractor pair. Specifically, the featural distances between the response /e/ and distractors /e/, /i/, /o/, and /u/ are 0, 1, 2, and 3, respectively. Similarly, the featural distances between the response /u/ and distractors /u/, /o/, /i/, and /e/ are 0, 1, 2, and 3, respectively. Our hypothesis was that a smaller featural distance (number of feature differences) speeds up RTs relative to a greater featural distance.

**Table 2:** Featural distance between vowels used as distractors and cued responses based on standard phonological representations

Distractor	Features				Cued response	
					Distance from /e/	Distance from /u/
/e/	[−round]	[−back]	[−high]	[−low]	0	3
/i/	[−round]	[−back]	[+high]	[−low]	1	2
/o/	[+round]	[+back]	[−high]	[−low]	2	1
/u/	[+round]	[+back]	[+high]	[−low]	3	0

**Fig. 3:**
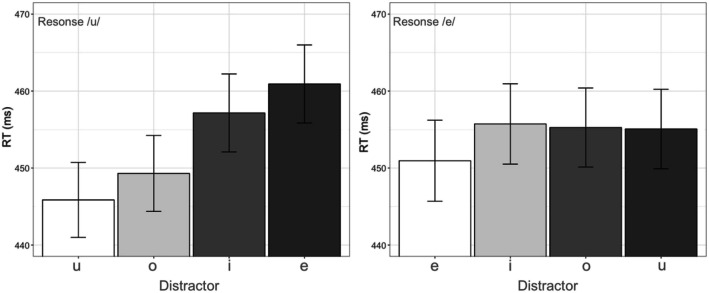
Mean RTs (ms) showing effects of each distractor vowel for the responses /u/ (left) and /e/ (right). Error bars show 95% confidence intervals. Results are collapsed across SOAs, because the interactions of SOA and distractor conditions were not significant

Figure 3 shows mean RTs (in ms) by featural distance across both responses. To test for the effect of featural distance, a model was fitted with featural distance (number of different features as shown in Table 2) entered as a continuous variable ranging from 0 (no difference) to 3 (a large difference). Response and the interaction between response and featural distance were included to examine the generalizability of any featural distance effect across responses. The interaction between SOA and featural distance also was included to examine the sensitivity of featural distance effects across SOAs. The random effects structure included by-participant slopes involving effects and interactions of featural distance, SOA and response as these were repeated across participants. A total of 16,409 trials were included in the model.^2^

Table 3 shows the results. The distractor with “zero” featural distance was the baseline (intercept) condition, meaning that coefficients for featural distance are relative to a distractor phonemically identical to the cued vowel. RTs were significantly longer (3.32 ms) with each unit increase of featural distance between the cued response and distractor (|*t*| = 4.15). All nonlinguistic effects were significant in line with their effects in the previous analysis. The effect of response was not significant, but the interaction of featural distance and response was significant (|*t*| = 3.11). While the effect of SOA was significant, its interaction with featural distance was not (|*t*| < 0.5).

**Table 3:**
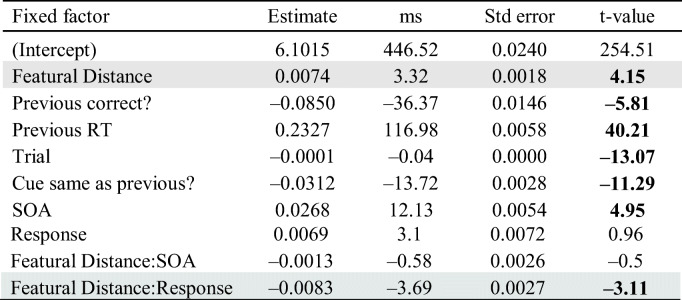
Results of a linear mixed-effect model of log RTs (featural distance effect)

*Note*. The effect of featural distance compared with the “zero” condition and the interaction between featural distance and response are shaded. Boldface *t* values indicate significant predictors in the model (|*t*| > 2)

The significant interaction between featural distance and response indicates a different pattern of distractor effects on the responses /e/ and /u/. Figure 3 shows the mean RTs for each distractor separately for the responses /e/ and /u/.

To elucidate the interaction between featural distance and response, the data for each response were analyzed separately to examine effects of featural distance on each response. For both models, the fixed and random effects were identical to the previous model, except that Response and Featural Distance × Response interaction was removed from these models (since each model contained data from only one response). The effect of featural distance was only significant for response /u/ (|*t*| > 4.47), but not for response /e/ (|*t*| = 0.89).

Further, in a set of separate analyses for each response, we tested the effect of each response–distractor combination. Tables 4 and 5 show the results from the models fitted to the data for response /u/ and /e/, respectively. This time distractor (four levels: /i/, /e/, /o/, /u/) was used as a categorical fixed factor instead of the continuous measure of featural distance, and the identical response–distractor condition served as the baseline. RTs for distractor /e/ and /i/ (whose featural distance from response /u/ is 3 and 2, respectively) were significantly longer compared with the condition where response and distractor were both /u/ (featural distance is 0). However, there was no significant effect for distractor /o/ (|*t*| = 1.17); that is, for distractor /o/, the vowel whose featural distance from response /u/ was 1 (as opposed to 2 and 3 for /i/ and /e/), RTs were not significantly different from the baseline. All nonlinguistic effects were significant. The effect of SOA was not significant, nor were the interactions between SOA and the various distractors (all |*t*| < 1.58).

**Table 4:**
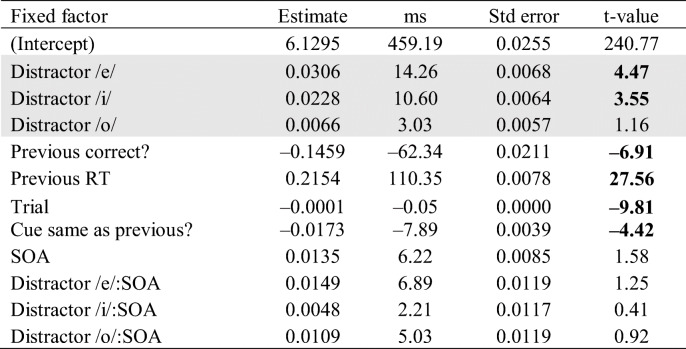
Results of a linear mixed-effect model of log RTs (effect of different distractors − response /u/)

*Note*. The effect of the different distractors is compared with the condition where response and distractor were both /u/. The distractor conditions are shown in the shaded rows. Boldface *t* values indicate significant predictors in the model (|*t*| > 2)

**Table 5:**
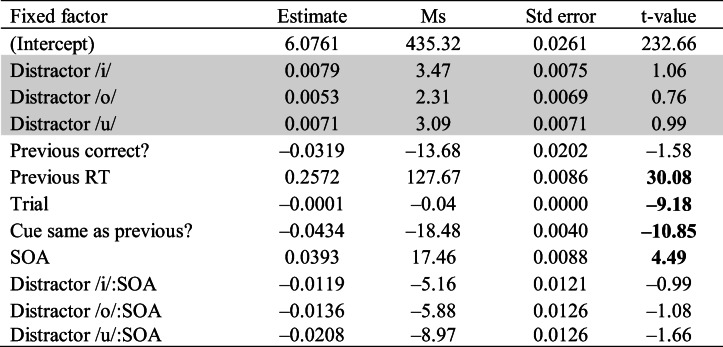
Results of a linear mixed-effect model of log RTs (effect of different distractors − response /e/)

*Note*. The effect of the different distractors is compared with the condition where response–distractor were both /e/. The distractor conditions are shown in the shaded rows. Boldface *t* values indicate significant predictors in the model (|*t*| > 2)

Table 5 shows the results for response /e/. None of the distractors resulted in RTs significantly longer than the phonemically identical distractor /e/. All nonlinguistic effects were significant. The effect of SOA was significant. However, the interactions between SOA and the various distractors were not (all |*t*| < 1.66).

Comparison of the patterns of results for the two responses (/e/ and /u/) reveals that, whereas there are modulation of RTs depending on the distractor for the response /u/, there are no such modulation effects for any of the distractors /i/, /o/, or /u/ when the response was /e/ (*|t|* < 1.06). Further, for the response /u/, delays due to the distractors /e/ and /i/ only are significant (*|t|* > 4.47). We take up these differences in RT modulations between the two responses in the following section.

## Discussion

We addressed how the speech production and perception systems interact during online processing of vowels by using an experimental task which requires concurrent use of both systems. During the cue–distractor task, participants repeatedly produce responses prompted by a visual cue. Shortly after presentation of the cue but before any response is given, participants hear a distractor syllable via headphones. By systematically manipulating the relation between the response and the distractor, this task has been used extensively to demonstrate perceptuomotor integration effects (that is, perception effects on production) mainly with consonants: Response times speed up when the distractor syllable begins with a consonant that shares properties (such as articulator or voicing) with the initial consonant of response compared with conditions when properties are not shared. Here it is demonstrated that perceptuomotor integration is not limited to consonants and that congruency effects go beyond phonemic (non-)identity also for vowels.

Our first hypothesis was that phonemically congruent response–distractor pairs of vowels (e.g., /e/–/e/ or /u/–/u/) will result in faster RTs than phonemically incongruent pairs (e.g., /e/–/u/ or /u/–/i/), as has previously been reported for consonants (Galantucci et al., 2009; Kerzel & Bekkering, 2000) and in one recent study also for vowels (Adank et al., 2018). This hypothesis was confirmed. We found that distractor vowels that are phonemically different from the response vowel resulted in RTs that were on average 6.94 ms slower than distractor vowels that are phonemically identical. This first assessment of perceptuomotor effects for vowels does not consider the subphonemic properties of the vowels in the response–distractor vowel pairs because all phonemically different (from the response) distractor vowels were collapsed into a single group per spoken response. Our second hypothesis was that distractor vowels which are subphonemically more like the response vowel will speed up RTs compared with distractor vowels which are subphonemically less like the response. We defined subphonemic similarity in vowel pairs by referring to featural distance (i.e., the number of phonological features that differ between the two vowels in each pair). Extending earlier findings for consonants (Roon & Gafos, 2015) to the case of vowels, we observed that greater featural distance between the response and distractor resulted in slower RTs (by approximately 3.32 ms per unit of featural distance) compared with when the response and distractor are phonemically the same. Table 6 summarizes mean RT delays for each incongruent response–distractor vowel pair relative to the corresponding congruent response–distractor pair as estimated by our models.

**Table 6:** Perceptuomotor interference effects for each response–distractor vowel pair

Response /u/		Response /e/	
Distractor /e/	14.26 ms	Distractor /u/	3.09 ms
Distractor /i/	10.60 ms	Distractor /i/	3.47 ms
Distractor /o/	3.03 ms	Distractor /o/	2.31 ms

Comparing the present results to previous studies using the cue–distractor task, it appears that the interference effect—the temporal delay in giving a spoken response—is typically larger for consonants. For example, Galantucci et al. (2009) report an average effect of 28 ms. Our results uncovered an average interference effect about 7 ms for phonemic incongruency (see Table 1). This is in line with the results reported by Adank et al. (2018) for vowels embedded in a consonant frame (13 ms for their Experiment 1 and 7 ms for their Experiment 2; note that these values include audio, visual and audiovisual modalities).

A possible explanation for the generally smaller delays in RTs observed for vowels compared with consonants may be due to differences in the granularity of the representations activated during speech perception in contrasts involved among vowels versus those involved among consonants. Across Galantucci et al.’s (2009) three experiments on consonants, responses and distractors always shared the same main articulator on congruent trials and differed on incongruent trials (e.g., the lips for /pa/, /ba/, and /ma/ vs. tongue tip for /ta/, /da/, and /na/). In another cue–distractor study, Klein et al. (2015) report RTs in which the responses and distractors shared (e.g., /da–ta/) or differed in the main articulator (/ka/–/ta/); additionally, the distractor’s voice onset time (VOT) was shorter or longer than the participant’s own VOT. While RTs were slower on trials when the articulator of the response was different from that of the distractor (compared with trials which shared the main articulator), RTs did not differ between trials where distractor VOTs were different (e.g., /ka/ with a longer VOT) relative to the participant’s mean VOT (e.g., /ka/ with a VOT shorter than that of the distractor). Likewise, Roon and Gafos (2015) found a small (4 ms) but nonsignificant effect when responses and distractors differed in voicing compared with no difference in voicing (e.g., /ta/–/ba/ vs. /da/–/ba/). While previous results show clear effects on RTs due to the distractor consonant’s articulator (Galantucci et al., 2009), this is not necessarily the case for VOT or voicing differences (Klein et al., 2015; Roon & Gafos, 2015). A plausible interpretation of the divergent effects of VOT/voicing and articulator in consonants, which bears on the case of vowels, may relate to possible differences in the somatomotor representations activated during speech perception of these different contrasts in terms of voicing versus main articulator. Suppose the response is /ba/ and the distractor is /da/. Planning to say /ba/ activates motor codes for the lips and hearing /da/ activates motor codes for a different main articulator (namely, the tongue tip). By contrast, planning to say /ka/ with a long VOT activates motor codes for the tongue dorsum and hearing /ka/ with a shorter VOT activates motor codes for exactly the same main articulator. In the case of vowels, differences between the response and distractor always involve the same articulators (lips and tongue). However, these articulators are used in different ways across different vowels—for example, planning to say /u/ actives motor codes for the lips and tongue and hearing /i/ also activates motor codes for the lips and tongue but, importantly, with different configurations. It is conceivable that the smaller delays in RTs for vowels (compared with stop consonant pairs like /ba/–/da/) are because similar but not identical motor codes (rather than motor codes for distinct articulators) are involved across the response and distractor on incongruent trials. Adank et al. (2018) suggest that future studies should investigate this very issue by comparing the articulatory complexity of different sounds via exploring the somatotopy of perceived sounds using transcranial magnetic stimulation (TMS) and measuring motor evoked potentials (MEPs) from lip and tongue muscles.

Our results raise new questions about how the substance underlying phonemic categories is to be described, because perceptuomotor effects were evidently not uniform across the two responses. Recall that our second model revealed a significant interaction of featural distance and response. Subsequent analyses showed that, whereas RT modulations for the response /u/ depended on the identity of the distractor, RTs for the response /e/ were *not* reliably affected by the exact distractor vowel (/i/, /o/ or /u/). Thus, there are different patterns of perceptuomotor effects for the /e/ and /u/ responses.

Why might perceptuomotor effects occur for the response /u/, but not for /e/? To define featural distance, we followed convention by using the standard phonological representations for vowels that assume that all features of every vowel are specified. An alternative to this approach to featural representations is Lahiri and Reetz’s (2010) featurally underspecified lexicon model (FUL). In this model, representations may be underspecified for certain features. For instance, the feature [coronal], originally used to represent sounds produced with a raised tongue tip blade (Chomsky & Halle, 1968, p. 304), but adopted in Lahiri and Reetz’s (2010) set of features to also represent front vowels, is claimed not be specified in mental representations. Thus, while the representation for /e/ lacks feature values for coronality and height, the representation for /u/ is fully specified ([dorsal], [labial] and [high]). According to FUL (Lahiri & Reetz, 2010), in perceiving speech, the underspecified features of a phoneme are extracted from the incoming signal and these ‘surface’ features may or may not mismatch with another phoneme’s mental representations. In the present study, the features in the mental representations of the response vowels may or may not mismatch with the surface features extracted from perceiving a distractor vowel. For instance, when the response is /e/ and the distractor is /u/, the features of the response vowel /e/ will not mismatch with any of the surface features extracted from perceiving the incoming distractor /u/ (namely, [dorsal], [labial], [high]) because the mental representation of /e/ has no specification for any of these features. On the other hand, when the response is /u/ and the distractor is /e/, there is a mismatch between the [dorsal] feature in /u/’s mental representation and the [coronal] feature extracted from the incoming distractor /e/. Table 7 shows a schematic of the relationships between the features for the mental representations of the two response vowels /e/ and /u/ and the features extracted from perceiving different distractor vowels.

**Table 7:**
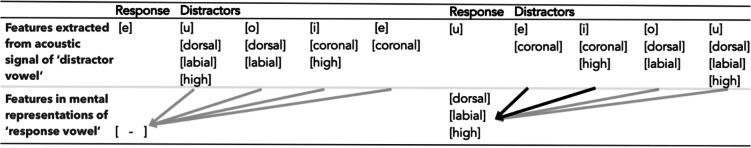
Phonological features extracted from the perceived incoming signal (surface form) and those in the mental representation (for production of the response) based on an alternative model of representations admitting underspecification (Lahiri & Reetz, 2010)

*Note*. Arrows represent the relationship between features extracted form distractors /u/, /o/, /i/, /e/ for conditions where the response was /e/ or /u/. Grey arrows indicate pairs of distractor and response vowels in no-mismatch conditions, and black arrows indicate pairs in mismatch conditions

Our results are compatible with the predictions of the underspecified representations model; whereas we found a modulation of RTs depending on the distractor for the response /u/, no such modulation effects were found for any of the distractors /i/, /o/, or /u/ when the response was /e/. The absence of such RT modulations for response /e/ may be seen as a consequence of the lack of mismatch between response /e/ and any of the incoming distractor vowels as shown in Table 7. In contrast, for response /u/, its mental representation is specified for [dorsal], [labial], and [high]. Consequently, hearing the distractors /e/ or /i/ while planning to produce the response /u/ would result in a mismatch between /u/’s [dorsal] representation and the surface [coronal] feature of the distractors /e/ or /i/. This is in line with the results indicating significant effects (delays in RTs) for the distractors /e/ and /i/ when the response was /u/. Additionally, FUL predicts no mismatch between the underlying features of the response /u/ and the surface features extracted from the distractor /o/. This is because the distractor /o/ exhibits [dorsal] and [labial], which are also exhibited in the mental representation of the response /u/. In line with this, the results showed no significant difference in RTs when the response was /u/ and the distractor was /o/ compared with when the response and distractor were both /u/.

While our results show that previous findings for consonants about phonemic and subphonemic properties influencing perceptuomotor effects also apply in the case of vowels, the design of the present study differs in one notable way from previous cue–distractor studies. In this study, the required responses and the distractors were isolated vowels (e.g., /e/ or /u/) instead of more linguistically typical syllables containing consonant–vowel (CV) or consonant–vowel–consonant (CVC) phoneme sequences (e.g., /ba/ or *heed*). Note that stop consonants like [b] and [d] cannot form (acoustic) stimuli in isolation (due to the lack of acoustic output from a fully constricted vocal tract) whereas vowels can. In some sense, our design decision of using isolated vowels provides the simplest test bed for assessing perceptuomotor effects, and such a test bed is not available for consonants; all previous perceptuomotor studies for consonants use the simplest possible form—namely, a consonant–vowel syllable where the vowel is kept the same across the differing with respect to the consonant stimuli. However, our design decision for vowels may not be optimal in a different sense. In vowel perception, identification accuracy is substantially reduced for isolated vowels which have been excised from coarticulated CVC syllables compared with vowels in intact CVC syllables (for a review, see Strange & Jenkins, 2013). Strange and Jenkins (2013) hypothesize that the dynamic time-varying spectral structure found in the edges of vowels in coarticulated syllables is rich in information for perceiving phonemic identity in vowels. Recall that our distractor stimuli were isolated vowels excised from CVC syllables which excluded much of the spectrotemporal structure around the edges of the vowels. It is thus possible that, if the distractor vowels had been presented in the original CVC syllables (and the required responses were also CVC syllables), modulations in RTs due to the (in)congruency of response–distractor pairs would have been more pronounced than those observed, potentially due to the availability of spectrotemporal cues relevant for perceiving phonemic identity which is enhanced in CVC syllables. In future studies, we thus plan to examine RT modulations with vowels in CVC syllables. Additionally, while the findings of this study seem compatible with featural representations, our study was not designed to address whether a metric of similarity based on acoustic parameters versus phonological features best accounts for the observed RT modulations. A major challenge in separating feature-based from acoustic-based notions of similarity is that featural congruency often correlates with acoustic similarity. In the general case, the more features that differ between two vowels, the more different the vowels are acoustically. Teasing apart the two notions of similarity requires comparing perceptuomotor effects for acoustically similar but featurally different vowels with featurally similar but acoustically different vowels. We plan to undertake such comparisons by employing languages with vowel inventories which include such contrasting pairs in future studies.

Let us address next implications of our results about speech perception and production models. The findings of our study necessitate a formulation of the perception–production link where motor codes activated in the process of planning a response vowel are also activated automatically by a perceived distractor vowel. According to the motor theory of speech perception (Liberman & Mattingly, 1985), these motor codes are the sole object of perception, and our results are therefore fully compatible with that theory. However, our results do not entail that the codes activated in perception must exclusively be motor codes and our study was not designed to address whether in perception non-motor codes are also activated (cf. Diehl, Lotto, & Holt, 2004; Galantucci, Fowler, & Turvey, 2006). Thus, our results are compatible with theories in which nonmotor codes are also activated during perception (e.g., Fowler, 1986; Mitterer & Ernestus, 2008; Ohala, 1996), provided some link between the nonmotor and motor codes can be assumed (Viviani, 2002). In terms of speech production models, our findings of RT modulations due to subphonemic properties are more in line with speech production models which assign roles for subphonemic parameters in the process of speech production (e.g., Dell et al., 1993) compared with other theories which do not assign important roles for subphonemic features in the process of planning articulation (e.g., Roelofs, 1997).

In summary, using an experimental paradigm which requires concurrent use of the perception and production systems, we studied perceptuomotor integration effects—that is, how perception of an auditory vowel stimulus (the distractor) affects production of a cued vowel response. Our results contribute to and extend previous perceptuomotor studies focusing on consonants (Galantucci et al., 2009; Kerzel & Bekkering, 2000; Roon & Gafos, 2015). In line with previous studies on consonants using the cue–distractor task, we found that RTs for producing a required vowel response speed up when the distractor vowel is phonemically identical to the cued spoken response compared with when the distractor is phonemically different. We also found evidence that subphonemic properties – below the level of phonemic category—modulate RTs. The fact that participants in our experiments were told to ignore the distractor and yet reliable effects of distractors on responses are observed, both in our current study and previous studies using the same paradigm (e.g., Adank et al., 2018; Galantucci et al., 2009; Roon & Gafos, 2015), attests to the automaticity of the perceptuomotor effects and to the promise of this paradigm in elucidating the perception–production link for vowels as well as for consonants. In future studies, we plan to employ more speech-typical utterances with vowels embedded in syllables (as opposed to isolated vowels) as well as assess the extent to which other similarity metrics based on acoustic dimensions (as opposed to phonological features) provide a better basis for congruency relations giving rise to perceptuomotor integration effects.

## Footnotes

1 Trials from the no-distractor condition (4,560 trials) were excluded because by definition they are not relevant in comparing response–distractor effects and, of course, have no SOAs, as SOA is the lag between the onset of the visual cue and the presentation of the distractor.

2 The tone and no-distractor conditions were excluded from the analysis as these clearly cannot be defined by referring to the number of phonological feature differences.
